# Coevolution of amino acid residues in the key photosynthetic enzyme Rubisco

**DOI:** 10.1186/1471-2148-11-266

**Published:** 2011-09-23

**Authors:** Mingcong Wang, Maxim V Kapralov, Maria Anisimova

**Affiliations:** 1 Computational Biochemistry Research Group, Department of Computer Science, Swiss Federal Institute of Technology (ETH), Zurich, Switzerland; 2 Swiss Institute of Bioinformatics (SIB), Switzerland; 3 Department of Plant Sciences, University of Oxford, Oxford, UK; 4 Institute of Molecular Life Sciences, University of Zurich, Switzerland

**Keywords:** Rubisco, coevolution, phylogeny, positive selection

## Abstract

**Background:**

One of the key forces shaping proteins is coevolution of amino acid residues. Knowing which residues coevolve in a particular protein may facilitate our understanding of protein evolution, structure and function, and help to identify substitutions that may lead to desired changes in enzyme kinetics. Rubisco, the most abundant enzyme in biosphere, plays an essential role in the process of carbon fixation through photosynthesis, thus facilitating life on Earth. This makes Rubisco an important model system for studying the dynamics of protein fitness optimization on the evolutionary landscape. In this study we investigated the selective and coevolutionary forces acting on large subunit of land plants Rubisco using Markov models of codon substitution and clustering approaches applied to amino acid substitution histories.

**Results:**

We found that both selection and coevolution shape Rubisco, and that positively selected and coevolving residues have their specifically favored amino acid composition and pairing preference. The mapping of these residues on the known Rubisco tertiary structures showed that the coevolving residues tend to be in closer proximity with each other compared to the background, while positively selected residues tend to be further away from each other. This study also reveals that the residues under positive selection or coevolutionary force are located within functionally important regions and that some residues are targets of both positive selection and coevolution at the same time.

**Conclusion:**

Our results demonstrate that coevolution of residues is common in Rubisco of land plants and that there is an overlap between coevolving and positively selected residues. Knowledge of which Rubisco residues are coevolving and positively selected could be used for further work on structural modeling and identification of substitutions that may be changed in order to improve efficiency of this important enzyme in crops.

## Background

Coevolution is one of the few paramount forces acting on all levels of biological organization from bioms to nucleotides. Observations of the complementary adaptations in two or more species caused by mutual selection pressures have started from Darwin's (1862) work on orchids and their pollinators and resulted in theoretical generalizations such as 'Red Queen Hypothesis' [1,2]. More recently concepts and methodologies developed for the study of species coevolution were applied to the growing wealth of molecular data, in particular for detection of coevolution between and within proteins [3]. Identifying coevolving positions in proteins allows better understanding of their structure and function and paves the road to engineering proteins with desired properties. Several computational methods have been proposed to detect coevolving residues from multiple sequence alignments (e.g., [4-8]). Best approaches strive to disentangle patterns created by coevolution and those due to shared ancestry (phylogenetic correlation) and stochasticity (random error). Based on recent comparative evaluation of the state-of-art techniques to detect coevolution [9], here we use one of the top performing approaches implemented in CoMap [6] to study the coevolution of residues in a key photosynthetic enzyme Rubisco.

Rubisco (ribulose-1,5-bisphosphate carboxylase/oxygenase, EC 4.1.1.39) is the key enzyme of the Calvin cycle, catalyzing the fixation of inorganic carbon dioxide to organic sugars. Rubisco is a gateway for inorganic carbon, which is present in all light-dependent ecosystems. However, due to the poor turnover rate and competition between O_2 _and CO_2 _at the active site, Rubisco is often the rate-limiting step of the photosynthesis [10]. These properties of Rubisco coupled with its high concentration in photosynthesizing organs make it the most abundant enzyme on Earth [11]. Both biospheric importance and intracellular abundance of Rubisco stimulated plenitude of molecular studies using Rubisco as a model system (reviewed in [12]), but despite significant progress our understanding of Rubisco functioning and evolution is still far from complete [13].

Land plants and green algae have type IB Rubisco, which is a hexadecamer consisting of eight large, plastid encoded, and eight small, nuclear encoded, subunits. Large subunits which possess the active site of Rubisco are encoded by the chloroplast gene *rbcL*, which over three decades ago was among the first fully sequenced genes [14] and since that time became one of the most often sequenced genes thanks to its wide use in phylogenetics of plants and algae (e.g. [15]). Plant systematists have mainly used *rbcL *paying little attention to its function. However, recently positive selection acting on *rbcL *was found in the most lineages of land plants [16]. The mapping of the positively selected residues on Rubisco tertiary structure revealed that they are located in regions important for dimer-dimer, intradimer, large subunit-small subunit and Rubisco-Rubisco activase interactions, and that some of the positively selected residues are close to the active site [16]. Positive selection on Rubisco is in concert with well-known variation in Rubisco kinetics found in different species (e.g. [17]) and its correlation with environmental parameters (e.g. [18]). Positive selection has been shown as a driving force for kinetic differences in Rubiscos of C_3 _and C_4 _plants [19,20].

Coevolutionary studies have been applied to a few important proteins and provided new information about protein-protein interactions, ligand-receptor bindings, and the 3D protein structure [8,21-23]. Here we study the coevolution and positive selection on Rubisco large subunit using 142 data sets of the *rbcL *gene representing the main lineages of land plants (for detailed description see [16]). Our aim is to provide a better insight into the patterns of groups of non-independent sites and positively selected sites as well as to find their amino acid composition, pairing preference, and spatial distribution.

## Results and discussion

### About half of Rubisco residues coevolve

In total 237 groups of residues were detected as coevolving for different amino acid properties: 26 groups for charge, 71 for the Grantham distance, 80 for polarity, and 60 for volume. No groups with compensatory changes were detected. The identified coevolving residues clustered in groups of 2 - 16 residues, and were widely distributed across the sequence. Around 50% (237 out of 476) of the large subunit residues were involved in coevolution. Most of them were involved in the subtle changes of the biochemical properties of its surrounding structure, whereas there were 54 residues located within the structurally and/or functionally important sites (Table 1). The proportions of the coevolving residues among sites involved in structural and/or functional interactions and among the rest of sites were 22.8% and 21.8%, respectively, and did not differ significantly. Among these 54 coevolving sites, 25 were involved in the dimerization of the two large subunits, 16 residues were important for the dimer-dimer associations and 19 of them were found to be important for the interaction between the large and small subunits (Table 1).

**Table 1:** Known interactions of the inferred coevolving residues

Interactions	Residue no
Intradimer (ID)	15, 63, 64, 106, 109, 121, 126, 128, 129, 131, 176, 180, 205, 207, 208, 209, 211, 271, 297, 408, 413, 461
Dimer-Dimer (DD)	34, 105, 142**, 143, 146, 147, 162, 164, 216, 249, 285, 286
Small Subunit (SSU)	76, 163, 166, 223, 226, 227, 229, 230, 260, 261*, 397, 433, 453, 454
DD and ID	210
SSU and DD	219, 258, 288
SSU and ID	74, 412

The residue number is according to the spinach Rubisco sequence (8RUC); * positively selected site; ** most often positively selected site; underlined residues coevolve with sites under positive selection; interactions are after Knight *et.al*. (1990).

**Figure 1:**
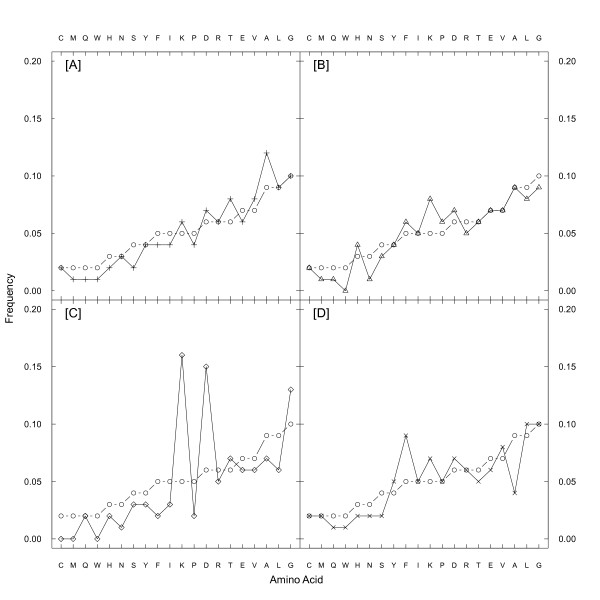
**The amino acid composition of residues inferred as coevolving for different biochemical properties: (A) polarity, (B) Grantham distance, (C) charge and (D) volume, as shown by symbols "plus", "triangle", "rhombus" and "cross', respectively**. The amino acids are ordered according to their frequency in all RBCL sequences, (as shown by "circle").

**Figure 2:**
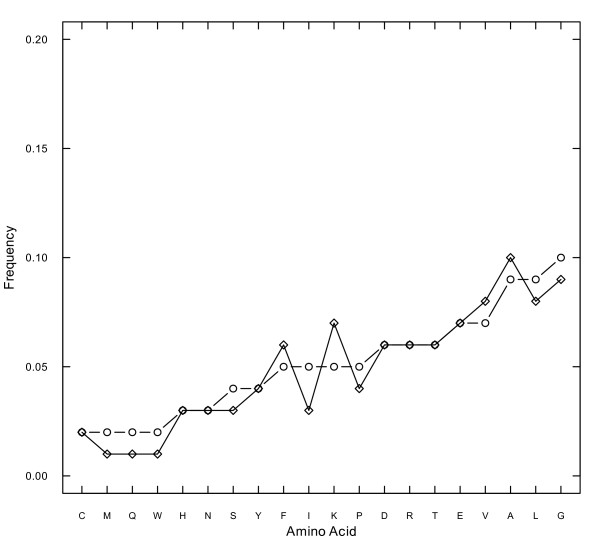
**The amino acid composition of all inferred coevolving sites (marked with "rhombus"), as compared to all RBCL sequences (marked with "circle")**. The amino acids are ordered according to their frequency in all RBCL sequences.

To test whether amino acid composition of coevolving sites is different from the whole sequence of the large subunit (LSU) of Rubisco, we performed the χ^2^-test for independence on the counts of amino acids in the two groups of sites. This test was highly significant for all four types (charge, volume, polarity and Grantham distance) of coevolving groups (*p*-values < 10^-15^). We further calculated the correlation of amino acid frequencies at coevolving sites as compared with frequencies found in the whole sequence. There were 1,396,945 residues in all data sets and 10,128 residues were shown to be coevolving for one or several biochemical properties. Among coevolving positions 1,714 residues were detected to be coevolving for charge, 7,087 residues for polarity, 4,550 residues for volume, and 5,272 residues were coevolving to conserve Grantham distance. The correlation coefficients *R *between the amino acid composition in the whole data set and the residues coevolving for charge, polarity, volume, Grantham and total (Figures 1, 2) were 0.63, 0.95, 0.80, 0.91, and 0.94, respectively. Thus, the residue composition of the sites coevolving for charge was the most different from the other regions of the protein, with the correlation of *R *= 0.63, which was lower than a threshold of 0.8 estimated in [24]. Meanwhile, for sites coevolving for other properties the residue composition was more similar to the composition of the whole protein (found *R *≥ 0.8), and also similar to the correlation between the composition of protein-protein interface residues and the composition of the whole protein [24].

Presumably, sites coevolving for certain biochemical properties (charge, volume, polarity and Grantham distance) may have different amino acid composition preferences. Thus, we studied how amino acid frequency was different in residues that coevolve compared to the whole sequences. All coevolving sites had higher proportion of A, C, E, F, K, V, T compared with whole sequences (Figure 2 and Additional file 1). Among these residues, A, C, F, V are hydrophobic and E, K, T are hydrophilic. Amino acids W, Q, M and I were underrepresented in all coevolving sites. Proportion of A, D, K, L, T and V were significantly higher in coevolving sites detected by polarity (Figure 1a). Proportion of D, F, H, K, P were significantly higher in coevolving sites detected by Grantham (Figure 1b). Proportion of K, D and G were significantly higher in coevolving sites detected by charge (Figure 1c) compared with whole sequences. Frequencies of D, F, K, L V, Y were significantly higher in sites coevolving for volume (Figure 1d). Thus, our results demonstrate that frequencies of certain amino acids at coevolving sites are significantly different from the amino acid composition found in the whole sequence.

**Figure 3:**
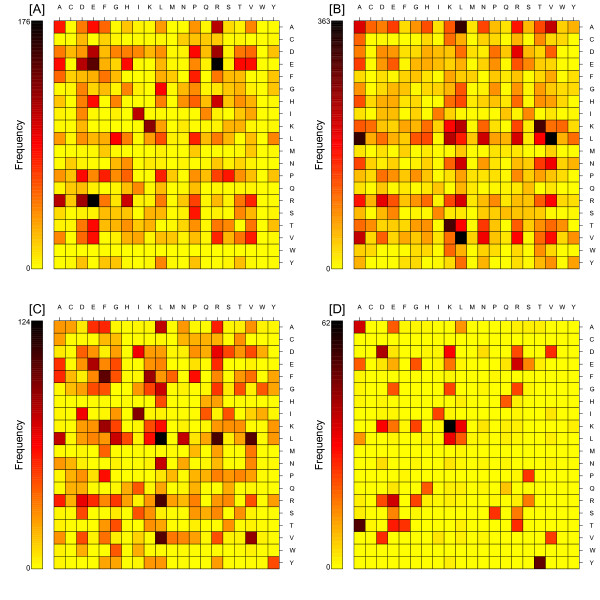
**The color-coded representation of the coevolution frequency matrix of amino acid pairs inferred coevolving with respect to different properties: (A) Grantham distance, (B) polarity, (C) volume, (D) charge**. The residues are arranged alphabetically.

Next, we estimated the residue-residue preference for inferred coevolving pairs: for each combination of 20 existing amino acids we counted how often a particular pair of amino acids *i *and *j *was inferred as coevolving in RBCL. For coevolving groups of more than two residues, all the pair-wise combinations were considered. The numbers of coevolving residue pairs are shown in the 20 × 20 symmetric matrices with respect to the four different amino acid properties and all the coevolving pairs (Figure 3.4). The most frequent entries of the matrices show which amino acid pairs most frequently coevolve. We observed that for coevolving sites, the residue-residue paring preference was different for each property (charge, volume, polarity and Grantham), probably due to specific biochemical constrains or interactions for each property type. For example, the residues with opposite charge, such as R-E, R-D, K-D and K-E, are often inferred as coevolving (Figure 3d). While pairs contenting the same charge are not very common, besides the pair K-K, which has extremely high frequency. Additionally, the charged residues also prefer to associate with small residues, such as T, G. Polar residues prefer to coevolve with other polar residues (e.g. K-T, R-D). Nonpolar residues on the other hand are more likely to coevolve with nonpolar residues (e.g. A-L, L-V, V-A) (Figure 3b). It seems that residues with similar volume tend to coevolve together more frequently (Figure 3c), such as L-L, F-F, I-I and V-L.

**Figure 4:**
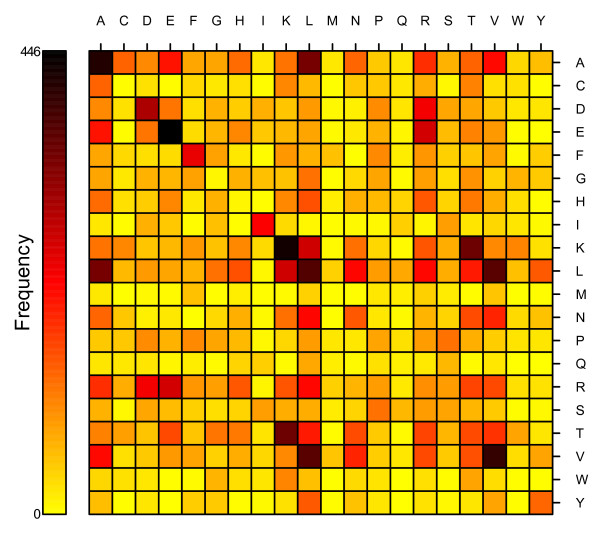
**The color-coded representation of the coevolution frequency matrix for all inferred coevolving pairs**. The residues are arranged alphabetically.

Of all the coevolving residue pairs, the hydrophobic pairs are most frequent compared with hydrophilic residues, such as A-A, A-L, I-I, L-L, L-V, F-F and V-V (Figure 4). This result is consistent with the previous study [24]. Nineteen of the coevolving residue pairs (out of total 400) appear to be responsible for more than 50% of the cases of coevolution. One possibility is that the evolutionary forces tend to be more similar for the similar amino acids, and when they evolve together, it makes it easier to keep the structure/environment stable.

### The amino acid composition of sites under positive selection is different from that of other sites

**Figure 5:**
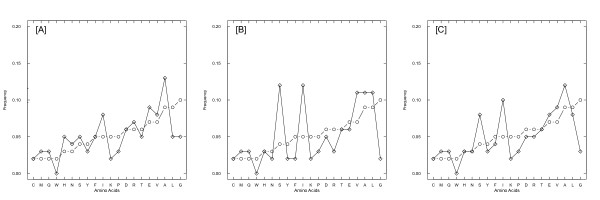
**The amino acid composition at the positively selected sites ("rhombus"), as inferred with (A) PAML, (B) FitModel, and (C) both PAML and FitModel**. The amino acids are ordered according to their frequency in all RBCL sequences (as shown by "circle").

Two types of models (implemented in PAML and FitModel) were used to detect sites under positive selection at the protein-coding level (see Methods for details). From the total 476 residues of the *rbcL *sequence, we detected 165 residues under positive selection using PAML, and 100 residues using FitModel (all with the posterior probability threshold of > 0.95). The correlation coefficients between the amino acid composition at positively selected sites and the whole sequences were 0.65, 0.47, 0.60 for residues detected with PAML, FitModel and with both, respectively (Figure 5). All the correlation coefficients were < 0.8, implying that the amino acid composition of sites under positive selection was quite different from that observed in the whole sequences of Rubisco's large subunit (see also Additional file 2). It appeared that preferred amino acids under positive selection were, either neutral hydrophobic, such as A, I, M and V or neutral polar, such as S and Q. While none of the hydrophilic residues were favored, such as W, K, G, the PAML results show that, the polar amino acids such as D, E, H, N were preferably located at the positive selected sites (Figure 5a). Interestingly the sites inferred to be under positive selection were not the same for PAML and FitModel. This may be explained by the differences in the formulations of the models used in each implementation. PAML analyses were conducted with site models that detect strong selective pressure affecting all lineages at specific sites. In contrast, the FitModel analyses were conducted with the model allowing switches between selective regimes through time, which therefore may detect sites under positive selection only at short time episodes (for review see [25]). For PAML analyses, the residues preferred to be under positive selection were A, D, E, H, I, M, N, Q, S, V (Figure 5a). For FitModel analyses, the residues preferred to be under positive selection were A, I, L, M, Q, S, V (Figure 5b). For sites detected with both PAML and FitModel, we observed a significant preference for amino acids A, I, M, Q, S, V (Figure 5c). It appears that the positive selection favored amino acids are either hydrophobic, such as A, I, M, V or polar, such as S, Q, but they are all neutral. While none of the hydrophilic residues are favored, such as W, K, G, the PAML results show that, the polar amino acids are preferably located on the positive selected sites, such as D, E, H, N.

The 14 residues most often inferred under positive selection were in accordance with the previous selection study of Rubisco [16]. Even though these residues are not directly located on the functional or structural important sites, they may be in contact with the active sites through dimer-dimer interaction, large subunits dimerization, and large and small subunit associations (Table 2). We studied whether any of the 14 most often positively selected residues were also involved in coevolution with other residues under positive selection. From Table 2 it can be seen that indeed some of sites under positive selection also coevolve with other sites under positive selection (e.g. 145&142, 142&255, 95&86 and 255&86).

**Table 2:** Fourteen of the most often positively selected residues of the Rubisco large subunit

Residue no^1^	Fitmode^2^	PAML^3^	Location of residues	Residues within 9 Å^4^	Coevolving residue no	Interactions
449	26	5	Helix G	374, 375, 376, 396, 399, 400, 401, 402, 407, 410, 411, 445, 446, 447, 448, 449,451, 452, 453, 454	128, 147	ID, SSU, DD
225	20	7	Helix 2	154, 155, 184, 187, 189, 219, 220, 221, 223, 224, 226, 227	None	DD, SSU, ID
251	20	4	Helix 3	209, 210, 213, 217,247, 248, 249, 250, 252, 253	258, 261*	DD, ID, SSU
145	16	7	Helix D	142**, 143, 144, 146, 147, 148, 149, 150, 281, 282, 314, 315, 316, 364, 365, 366	142**, 240	DD
142	14	4	Helix D	140,141, 143, 144, 145, 272, 276, 311, 312, 313, 314, 315, 364, 365	255**, 240	DD
95	13	4		23, 25, 26, 27, 54, 70, 71, 72, 73, 74, 93, 94, 96. 97. 98. 99	23, 86**, 326*, 332	SSU, ID
439	12	2	Helix G	413, 414, 417, 435, 436, 437, 438, 440, 441, 442, 443, 444, 446, 447	33*, 152, 151, 153, 135*, 281*, 310*, 440*, 470*	ID
219	11	4	Helix 2	180, 181, 182, 184, 185, 186, 215, 216, 217, 218, 220, 221, 222, 223, 224, 225,227	121, 388, 423	DD, SSU, ID
279	11	1	Helix 4	143, 144, 152, 249, 253, 254, 274, 285, 286, 277, 278, 280, 281	301, 346	DD
328	11	3	Loop 6	319, 320, 321, 322, 323, 324, 326, 327, 329, 330, 332, 333, 462, 464	228*, 281*	AS, ID
375	11	9	Strand 7	373, 374, 376, 377, 378, 379, 393, 396, 410, 411, 414, 436, 446, 449, 450, 453	395, 419*	AS, ID, SSU
255	9	6	Helix 3	190, 228, 229, 230, 231, 248, 253, 254, 256, 257, 280, 281, 282, 283, 315, 316	101, 86**, 167, 149*, 169*, 256*, 320*, 371*, 398	SSU
28	8	9	N-terminus	26, 27, 29, 30, 76, 91, 94, 128, 129, 130, 131, 132	19, 355*, 93*	SSU, ID
86	8	9	Strand C	33, 34, 35, 36, 37, 39, 41, 81, 84, 85, 87, 88	149*, 256*, 167, 169*, 222*, 317*, 320*, 371*, 398, 23, 30*	DD

^1 ^The residue number is according to the spinach Rubisco sequence (8RUC)

^2 ^Number of groups with detected positively selected residues in Fitmodel

^3 ^Number of groups with detected positively selected residues in PAML

^4 ^*positively selected site; ** most often positively selected site; underlined residues are both within 9 Å and coevolve

### The coevolving and positively selected sites are preferably located in helices

The protein secondary structure elements helix, strand and coil have different physical and chemical properties, thus play distinct roles in the protein tertiary structure and function. So the evolutionary force may vary among different secondary structures. In *Drosophila *proteins the coil regions are more likely to be under positive selection than expected, while the helices and strands undergo less positive selection [25].

The secondary structure of the large subunits of Rubisco is conserved throughout land plants, despite the variation in primary sequences [10]. The helix parts are usually amphipathic with one side hydrophobic and the other side hydrophilic, thus the structured regions can occur anywhere in the protein and involve the largest proportion of residues in Rubisco large subunit. The strands often contain hydrophobic residues and could form a well-structured parallel or anti-parallel beta sheet. The active site of Rubisco is located at the carboxy-terminal end of the beta strand [10]. The coil is the most flexible element without ordered structure and assists the conformational change of the protein. Loop 6 of Rubisco large subunit is conserved in land plants and green algae. It is crucial for the catalytic process because it controls the opened or closed state of the enzyme, which influences the association of the substrate [10]. It was shown that residues in mobile regions of the protein tend to evolve in highly correlated fashion, participating in physical and functional contacts during their motion [22].

**Figure 6:**
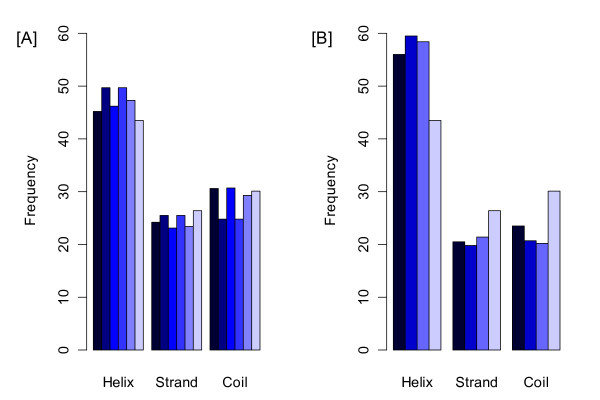
**Proportions of sites in different secondary structures: (A) color-coded bars from dark to light blue correspond respectively to the residues coevolving for Grantham distance, volume, charge, all coevolving sites and the whole RBCL sequence; (B) color-coded bars from dark to light blue represent respectively the positively selected sites detected by PAML, FitModel, both and the whole sequence**.

The study of the locations of coevolving residues of Rubisco with respect to the secondary structure could unravel the pattern of the coevolution at the structure level and explain how the different secondary structure elements may undergo different evolutionary forces. In plant Rubisco, the helix parts of the structure contain 47.3% coevolving residues (Figure 6a), which is significantly higher compared to 43.5% in the whole sequence. Moreover, helixes are enriched with sites coevolving with respect to all amino acid properties (polarity 45.7%, charge 46.2%, volume 49.7% and Grantham 45.2%). The coevolving residues in strands are fewer than in the whole sequence (coevolving 23.4%, total 26.4%). In coils the total coevolving residues are slightly less numerous than in the whole sequence (coevolving 29.3%, total 30.1%), but this trend changes for different properties. In coils the proportion of the sites coevolving for Grantham distance (30.6%) and charge (30.7%) are slightly higher compared to the whole sequence (30.1%). In light of widespread positive selection in plant Rubisco, the distribution of the positively selected sites in the secondary structure of Rubisco could suggest which parts of the structure are more sensitive to the selective forces. Interestingly, 58.4% of sites under positive selection were located in helices, which was significantly higher than compared to 43.5% among all sites (Figure 6b). The enrichment of helices with sites under positive selection was observed irrespectively to whether the sites were inferred with PAML or Fitmodel. Other parts of Rubisco structure contained fewer residues under positive selection compared to the whole sequence: in the strand regions 21.4% of sites were under positive selection compared to 26.4% overall, and in the coils 20.2% of sites were under positive selection compared to 30.1% overall.

Overall, this shows that evolutionary forces are unevenly distributed on the large subunit of Rubisco, with the helical parts of the structure more frequently affected by coevolution and positive selection compared to other parts. Interestingly, our results differ from ones obtained for *Drosophila *proteins where less than expected selection was found in helices [26].

### Coevolving residues are closer in 3D structure

In order to compare the distribution of physical distances between the coevolving residues and all the residues in the LSU of Rubisco, we used four known 3D structures of spinach and tobacco from PDB both in activated and non-activated states. For each PDB record, distances between the center masses of any two residues in the protein 3D structure were calculated. The coevolving residues were mapped onto the PDB structures, and all the pair-wise combinations of the coevolving sites within a group were listed. The corresponding distances of all the coevolving pairs were collected. The minimum pair-wise distances between residues for both the activated and unactivated state of each species were calculated and the smallest value was chosen for further comparisons. It is said that two residues are in physical contact, if the distance between them is under a certain threshold. In some studies, they use the distance between two beta carbons (Cβ) or two alpha carbons (Cα) of the corresponding residues, with the direct contact threshold of 8Å [22,23]. However, this method only considers one point of the residue, so that the possible position conformations of the other part of the molecule are neglected. In this study, distances between the center mass of the residues were calculated, thus the residue molecule was considered as a whole.

**Figure 7:**
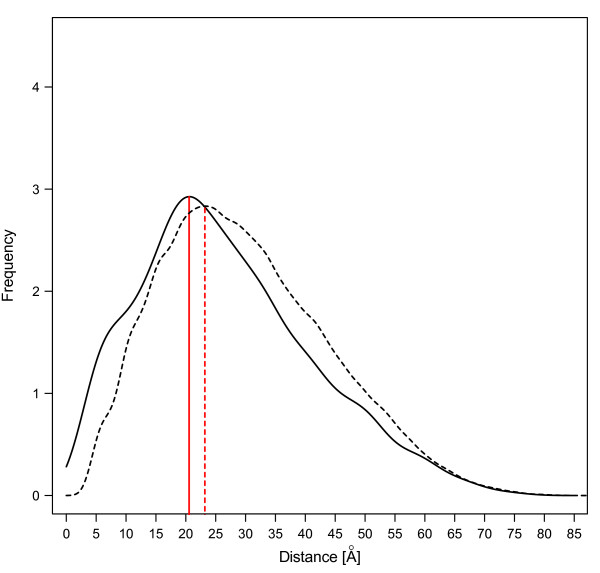
**The 3D distance distribution of the coevolving residue pairs compared to all residue pairs in Rubisco**. This is the minimum distance of the pair-wise residues between the active state and un-active state of Rubisco (based on the *Spinacia oleracea *structure 8RUC). Solid line is the distribution of pair-wise distances for coevolving residues. Dash line is the distribution of pair-wise distances for all protein residues.

The minimum physical distance between two coevolving sites in LSU varied from 3 Å to 70Å, with a mean value of 26.6Å. The one-sample Z-test was applied to the data set and showed that the average pair-wise distance between coevolving sites was significantly shorter than the average distance of the total pair-wise distance in one 3D protein chain (p < 0.01) (Table 3). Although some of the non-independent residues may be physically far from each other, long-term interactions through conformational changes and occurrence by chance [22] could indirectly lead to physical contact, such as, non-specific hydrophobic interaction. For coevolving residues we observed a clear shift to the left of the pairwise distance distribution compared to the distribution for the whole sequence (Figure 7). This suggests that on average pairs of coevolving sites in LSU are found closer in the 3D structure compared to the background.

**Table 3:** Physical distances between residue pairs

Species	Rubisco state	PDB	3D-distance			Coevolving sites distance				Positively selected sites distance				Positively selected site/active site distance			
		
			mean	median	**st.dev**.	mean	median	p-value^#^	Difference*	mean	median	p-value^#^	Difference*	mean	median	p-value^#^	Difference*
Nicotiana tabacum	Activated	4RUB	31.38	29.66	14.34	28.14	26.66	6.7E-9 (1.8E-8)	3.24	33.76	31.97	0.057 (0.036)	2.38	28.50	26.23	0.144 (0.164)	2.88
	
	Unactivated	1EJ7	31.44	29.86	14.24	27.5	25.6	5.6E-10 (6.9E-10)	3.94	33.01	32.40	0.146 (0.094)	1.57	27.97	25.28	0.099 (0.126)	3.47
	
	Average minimum		28.76	27.13	13.58	25.27	22.77	1.8E-8 (6.1E-9)	3.50	31.55	30.27	0.025 (0.019)	2.78	26.18	24.02	0.156 (0.192)	2.59

Spinacia oleracea	Activated	8RUC	31.48	29.77	14.37	28.21	26.56	1.1E-8 (1.3E-8)	3.27	34.15	32.08	0.038 (0.025)	2.62	28.74	26.38	0.156 (0.181)	2.74
	
	Unactivated	1RCX	31.24	29.54	14.26	27.97	26.23	8.5E-9 (1.1E-8)	3.27	33.97	32.12	0.034 (0.022)	2.73	28.51	26.09	0.155 (0.180)	2.73
	
	Average minimum		31.22	29.53	14.26	27.96	26.23	9.3E-9 (8.2E-9)	3.26	33.94	32.08	0.034 (0.023)	2.72	28.50	26.09	0.156 (0.180)	2.72

*The mean difference between the values of the 3D distance with the corresponding distance.

# p-values as calculated from one sample Z-test and, in parentheses, from the Wilcoxon Rank Sum Rank Test.

Next we analyzed physical proximity of the 14 residues most frequently found under positive selection in the LSU. Interestingly, these positively selected sites showed an opposite trend, as they were significantly further from each other than the background (Table 3), again showing different pattern from the *Drosophila *proteins [26]. The distances between active sites and sites under positive selection tended to be shorter compared with the background, although not significantly (possibly due to small samples).

## Conclusions

The functionally and structurally important sites in the protein are usually more conserved than other sites. But in some cases, one mutation at a crucial site may be compensated by mutations at other sites, so to maintain vital interactions and functions [22]. Our study shows that the coevolving and positively selected sites tend to be located within the functionally and structurally important regions of Rubisco. Substitutions have to be compatible with the protein function and be structurally stable. Therefore, the amino acid composition and the residues pairing preference of the sites under coevolution and positive selection can provide a better insight in terms of protein evolution. Our molecular evolutionary analysis reveals that different evolutionary forces may have distinct amino acid composition and pairing preferences. The coevolving residue composition may not be too different from that of the background because they are wide spread across the sequence, while the positive selection is quite different. Moreover, the groups of non-independent residues have their pairing preference. Based on the amino acid pairing frequency matrix with different biochemical properties, the distinct patterns of coevolving pairs could provide a hint for the further analysis about the mutual information.

Our study indicates that coevolving sites are in closer proximity in the tertiary structure of the Rubisco large subunit. Predicting protein tertiary structure from the primary sequence is a crucial problem in computational biology. The physical interactions between coevolving residues could help to build a residue contact map in protein tertiary structure analysis. Moreover, many residues which coevolve or under positive selection are found in the functionally or structurally important locations, such as dimer-dimer, intradimer, active site and small subunit interactions. Our results appear to be in agreement with the study of Yeang and Haussler [8], who proposed that in large protein families coevolving positions are spatially coupled and many of the coevolving positions are located at the functionally or structurally important positions. Furthermore, we find that many sites are both under positive selection and coevolution, suggesting that selection towards a new optimum may require more than one substitution. Indeed, multiple neutral changes along the mutational landscape of a protein may precede mutations with high advantageous fitness effect [27].

Because of the importance of Rubisco, it has been the target of genetic engineering for a long time. Aspects including structure, function and evolution of this enzyme have been studied with the aim to improve its kinetics. Nowadays, the experimental way of random mutagenesis and bioselection could be used to identify mutations that influence important properties of Rubisco [13,28-30], but the vast amount of candidates and the repetitive lab work make the process slow, unpredictable and tedious. Knowledge of location of coevolving or positively selected residues may be used to design future mutagenesis experiments and accelerate efforts to engineer better Rubisco, which would potentially increase the yield of agriculturally important crops.

## Methods

### 1. Sequence data and phylogeny estimation

We used 142 *rbcL *data sets from [16]. The sequences were assigned to each data set according to their phylogenetic relations. Each data set had 11 to 40 sequences. The codon alignments were constructed from DNA sequences by back-translating from amino acid alignments. Sequences within each data set were truncated to the same length. Angiosperms, gymnosperms, ferns, and mosses were represented by 122, 8, 9 and 4 datasets, respectively.

For each alignment a phylogeny was reconstructed using maximum likelihood as implemented in PhyML v3.0 [31,32]. During the inference we used amino acid models WAG [33] and LG [34], both with Γ-rate variation. The tree space was traversed using the combination of NNI and SPR heuristics [32]. The inferred phylogenies were used for further coevolution and positive selection analyses.

### 2. Coevolution Analysis

To detect coevolving residues we used a clustering approach that searches for ancestral co-substitutions or for compensatory changes by correlating amino acid substitution histories, as implemented in the R-program CoMap [6,35]. Substitution numbers for each branch were sampled from a posterior distribution based on a Markov substitution model and a phylogeny with branch lengths relating the sequences. Parameters of the models and tree branches were estimated by maximum likelihood prior to the sampling. Substitutions were weighted by different biochemical properties (charge, polarity, volume and Grantham) to detect coevolutionary trends specific to amino acid properties [7]. The amount of the biochemical change for one site was represented by weighted substitution vectors, containing weighted substitution counts for each branch of a phylogeny. The correlated or compensatory evolution was estimated based on the correlation coefficient of the substitution vectors. To select candidate groups, the complete linkage hierarchical clustering was applied to distance matrices based on the correlation and compensation statistics [6]. To asses the significance of inferred clusters, the parametric bootstrap with 1000 replicates was used to generate the joint null distribution of minimum site variability together with coevolution or compensation statistic *ρ*, as described in [6]. From such empirical distribution, *p*-values for each candidate cluster with observed statistic *ρ*_obs _were computed as Pr(*ρ *>*ρ*_obs _| *N*_min_), i.e., by conditioning on minimum site variability *N*_min _of the cluster. Clusters with *p*-value ≤ 0.05 were considered as evolving non-independently. A simulation procedure described in [6] was used to correct for multiple non-independent tests.

### 3. Positive Selection Analyses

Positive selection on the protein level was measured using the ω ratio, which is the ratio of nonsynonymous to synonymous substitution rates per site. Negative selection results in lower nonsynonymous rate relative to synonymous and so ω < 1. If the nonsynonymous mutations are favored, the nonsynonymous rate should be higher than synonymous, and so ω > 1 indicates evolution by positive selection. Here we estimated selective pressure on *rbcL *using different types of Markov models of codon evolution: (1) site-specific codon models that allow variation of selective pressure among sites in a sequence, and (2) switching codon models that allow variation of selective pressure among sites and over the evolutionary time. All branch lengths of inferred phylogenies were re-optimized under codon models.

#### 3.1. Detecting selection with site-specific codon models

Site-specific codon models were used to test each alignment for positive selection using likelihood ratio tests (LRTs) of nested models: M0 (one ratio) vs M3 (discrete), M1a (neutral) vs M2a (selection), and M7 (beta) vs M8 (beta & ω) [36]. These analyses were performed using the codeml program of the PAML package [37,38]. If the LRT for positive selection was significant, Bayes naïve empirical Bayesian approach was used to infer sites under positive selection [39]. Sites that were inferred to be under positive selection using model M8, if site's posterior probability for the positive selection class was ≥ 0.95.

#### 3.2. Detecting selective episodes with switching codon models

We also used switching Markov modulated codon models to detect episodes of positive selection during the evolution of Rubisco, as implemented in the program FitModel [40]. Each switching model used in our study allows three possible selective regimes for codon sites, for example like site model M2a with classes for positive (ω > 1), neutral (ω = 1), and negative selection (ω < 1), or like model M2 with 3 classes with no constraints on the ω ratio. Unlike site model, switching models allow each codon site to change the selective regime, and thus be affected by different selective pressures at different time points. This is accomplished by using an additional Markov process to describe the switches between selection regimes at any individual site. We used models both with bias (+S2) and with no bias (+S1) for switching between selective regimes. For each alignment we used LRTs to test whether switches of selective pressure over time occurred (M2a vs M2a+S1 and M3 vs M3+S1), and for alignments with significant evidence for switches we also tested whether there was switching bias (M2a+S1 vs M2a+S2 and M3+S1 vs M3+S2). Sites with episodes under positive selection were detected *a posteriori *using the Bayesian approach [40].

### 4. Mapping sites on 3D Structure

The analyses of location, properties and the distance analysis of residues in the protein structure were performed using VMD viewer [41], a program for visualization, manipulation and analysis of large molecules in three dimensions. Command options were used to extract information about sets of molecules, vectors and coordinates. The center mass of a molecule was computed using the Tcl language of VMD.

## Authors' contributions

MA conceived the study, and all three authors contributed to design of the study. MVK assembled the data. MA performed co-evolution inferences. MW performed all other analyses and statistical tests. All authors were involved in discussing, interpreting the results, and writing the manuscript. All authors read and approved the final manuscript.

## Supplementary Material

Additional file 1 Rubisco coevolving sites amino acid composition. Click here for file

Additional file 2 Positive selection amino acid composition. Click here for file
