# Supplementary material for: Coevolution of amino acid residues in the key photosynthetic enzyme Rubisco
Source: BMC Evol Biol. 2011 Sep 23;11:266. doi: 10.1186/1471-2148-11-266 (PMC3190394; doi:10.1186/1471-2148-11-266)
Supplement: Additional file 1 — Rubisco coevolving sites amino acid composition. [file 1471-2148-11-266-S1.DOC]

Appendix 1. Rubisco coevolving sites amino acid composition

|  |  |  |  |  |  |  |  |  |  |  |  |  |  |  |  |  |  |  |  |  |  |
| --- | --- | --- | --- | --- | --- | --- | --- | --- | --- | --- | --- | --- | --- | --- | --- | --- | --- | --- | --- | --- | --- |
|  |  |  |  |  |  |  |  |  |  |  |  |  |  |  |  |  |  |  |  |  |  |
| Amino acid | A | C | D | E | F | G | H | I | K | L | M | N | P | Q | R | S | T | V | W | Y | sum |
| Charge | 126 | 0 | 257 | 108 | 41 | 219 | 26 | 53 | 280 | 106 | 0 | 15 | 28 | 41 | 93 | 47 | 121 | 105 | 0 | 48 | 1714 |
|  | 0.07 | 0 | 0.15 | 0.06 | 0.02 | 0.13 | 0.02 | 0.03 | 0.16 | 0.06 | 0 | 0.01 | 0.02 | 0.02 | 0.05 | 0.03 | 0.07 | 0.06 | 0 | 0.03 | 1 |
| All-site | 130325 | 24840 | 79354 | 93762 | 62903 | 140860 | 45865 | 65027 | 65038 | 121055 | 28039 | 45652 | 63431 | 32321 | 89684 | 49433 | 88104 | 94874 | 22372 | 54006 | 1396945 |
|  | 0.09 | 0.02 | 0.06 | 0.07 | 0.05 | 0.1 | 0.03 | 0.05 | 0.05 | 0.09 | 0.02 | 0.03 | 0.05 | 0.02 | 0.06 | 0.04 | 0.06 | 0.07 | 0.02 | 0.04 | 1 |
| difference | -0.02 | -0.02 | 0.09 | -0.01 | -0.03 | 0.03 | -0.01 | -0.02 | 0.11 | -0.03 | -0.02 | -0.02 | -0.03 | 0 | -0.01 | -0.01 | 0.01 | -0.01 | -0.02 | -0.01 |  |
| difference ratio | -0.22 | -1 | 1.5 | -0.14 | -0.6 | 0.3 | -0.33 | -0.4 | 2.2 | -0.33 | -1 | -0.67 | -0.6 | 0 | -0.17 | -0.25 | 0.17 | -0.14 | -1 | -0.25 |  |
| P-value | 0 | 4.28E-008 | 2.20E-016 | 4.97E-001 | 2.52E-005 | 0 | 4.09E-005 | 2.14E-003 | 2.20E-016 | 0 | 5.26E-009 | 2.54E-008 | 7.49E-009 | 0.83 | 0.09 | 0.07 | 0.2 | 2.74E-001 | 2.16E-007 | 0.02 |  |
| significant | ** | ** | ** | no | ** | ** | ** | ** | ** | ** | ** | ** | ** | no | no | no | no | no | ** | * | ** |
|  |  |  |  |  |  |  |  |  |  |  |  |  |  |  |  |  |  |  |  |  |  |
| Grantham | 472 | 84 | 388 | 351 | 335 | 489 | 212 | 268 | 424 | 436 | 42 | 72 | 314 | 72 | 282 | 152 | 304 | 370 | 1 | 204 | 5272 |
|  | 0.09 | 0.02 | 0.07 | 0.07 | 0.06 | 0.09 | 0.04 | 0.05 | 0.08 | 0.08 | 0.01 | 0.01 | 0.06 | 0.01 | 0.05 | 0.03 | 0.06 | 0.07 | 0 | 0.04 | 1 |
| All-site | 130325 | 24840 | 79354 | 93762 | 62903 | 140860 | 45865 | 65027 | 65038 | 121055 | 28039 | 45652 | 63431 | 32321 | 89684 | 49433 | 88104 | 94874 | 22372 | 54006 | 1396945 |
|  | 0.09 | 0.02 | 0.06 | 0.07 | 0.05 | 0.1 | 0.03 | 0.05 | 0.05 | 0.09 | 0.02 | 0.03 | 0.05 | 0.02 | 0.06 | 0.04 | 0.06 | 0.07 | 0.02 | 0.04 | 1 |
| difference | 0 | 0 | 0.02 | 0 | 0.02 | -0.01 | 0.01 | 0 | 0.03 | 0 | -0.01 | -0.02 | 0.01 | -0.01 | -0.01 | -0.01 | -0.01 | 0 | -0.02 | 3.49E-005 |  |
| difference ratio | -0.04 | -0.1 | 0.3 | -0.01 | 0.41 | -0.08 | 0.22 | 0.09 | 0.73 | -0.05 | -0.6 | -0.58 | 0.31 | -0.41 | -0.17 | -0.19 | -0.09 | 0.03 | -0.99 | 0 |  |
| P-value | 0.35 | 0.31 | 1.49E-007 | 8.76E-001 | 1.01E-010 | 5.18E-002 | 0 | 1.41E-001 | 2.20E-016 | 3.08E-001 | 3.80E-010 | 8.35E-015 | 8.49E-007 | 4.80E-006 | 1.54E-003 | 0.01 | 0.11 | 0.51 | 2.20E-016 | 9.90E-001 |  |
| significant | no | no | ** | no | ** | no | ** | no | ** | no | ** | ** | ** | ** | ** | * | no | no | ** | no | ** |
|  |  |  |  |  |  |  |  |  |  |  |  |  |  |  |  |  |  |  |  |  |  |
| Polarity | 839 | 120 | 479 | 455 | 292 | 684 | 158 | 261 | 433 | 662 | 80 | 201 | 317 | 84 | 400 | 174 | 557 | 561 | 53 | 277 | 7087 |
|  | 0.12 | 0.02 | 0.07 | 0.06 | 0.04 | 0.1 | 0.02 | 0.04 | 0.06 | 0.09 | 0.01 | 0.03 | 0.04 | 0.01 | 0.06 | 0.02 | 0.08 | 0.08 | 0.01 | 0.04 |  |
| All-site | 130325 | 24840 | 79354 | 93762 | 62903 | 140860 | 45865 | 65027 | 65038 | 121055 | 28039 | 45652 | 63431 | 32321 | 89684 | 49433 | 88104 | 94874 | 22372 | 54006 | 1396945 |
|  | 0.09 | 0.02 | 0.06 | 0.07 | 0.05 | 0.1 | 0.03 | 0.05 | 0.05 | 0.09 | 0.02 | 0.03 | 0.05 | 0.02 | 0.06 | 0.04 | 0.06 | 0.07 | 0.02 | 0.04 | 1 |
| difference | 0.03 | 0 | 0.01 | 0 | 0 | 0 | -0.01 | -0.01 | 0.01 | 0.01 | -0.01 | 0 | 0 | -0.01 | -0.01 | -0.01 | 0.02 | 0.01 | -0.01 | 0 |  |
| difference ratio | 0.27 | -0.05 | 0.19 | -0.04 | -0.08 | -0.04 | -0.32 | -0.21 | 0.31 | 0.08 | -0.44 | -0.13 | -0.01 | -0.49 | -0.12 | -0.31 | 0.25 | 0.17 | -0.53 | 0.01 |  |
| P-value | 4.47E-013 | 0.59 | 9.23E-005 | 0.33 | 1.21E-001 | 2.28E-001 | 6.71E-007 | 1.06E-004 | 7.00E-009 | 0.04 | 1.40E-007 | 4.14E-002 | 0.78 | 2.78E-010 | 7.84E-003 | 8.31E-007 | 8.32E-008 | 1.76E-004 | 1.08E-008 | 8.53E-001 |  |
| significant | ** | no | ** | no | no | no | ** | ** | ** | * | ** | * | no | ** | ** | ** | ** | ** | ** | no |  |
|  |  |  |  |  |  |  |  |  |  |  |  |  |  |  |  |  |  |  |  |  |  |
| Volume | 169 | 85 | 336 | 291 | 417 | 436 | 89 | 231 | 339 | 463 | 103 | 99 | 206 | 66 | 284 | 86 | 207 | 370 | 46 | 227 | 4550 |
|  | 0.04 | 0.02 | 0.07 | 0.06 | 0.09 | 0.1 | 0.02 | 0.05 | 0.07 | 0.1 | 0.02 | 0.02 | 0.05 | 0.01 | 0.06 | 0.02 | 0.05 | 0.08 | 0.01 | 0.05 |  |
| All-site | 130325 | 24840 | 79354 | 93762 | 62903 | 140860 | 45865 | 65027 | 65038 | 121055 | 28039 | 45652 | 63431 | 32321 | 89684 | 49433 | 88104 | 94874 | 22372 | 54006 | 1396945 |
|  | 0.09 | 0.02 | 0.06 | 0.07 | 0.05 | 0.1 | 0.03 | 0.05 | 0.05 | 0.09 | 0.02 | 0.03 | 0.05 | 0.02 | 0.06 | 0.04 | 0.06 | 0.07 | 0.02 | 0.04 | 1 |
| difference | -0.06 | 0 | 0.02 | 0 | 0.05 | -0.01 | -0.01 | 0 | 0.03 | 0.02 | 0 | -0.01 | 0 | -0.01 | 0 | -0.02 | -0.02 | 0.01 | -0.01 | 0.01 |  |
| difference ratio | -0.6 | 0.05 | 0.3 | -0.05 | 1.04 | -0.05 | -0.4 | 0.09 | 0.6 | 0.17 | 0.13 | -0.33 | 0 | -0.37 | -0.03 | -0.47 | -0.28 | 0.2 | -0.37 | 0.29 |  |
| P-value | 2.20E-016 | 0.65 | 7.21E-007 | 0.39 | 2.20E-016 | 0.26 | 5.20E-007 | 1.77E-001 | 2.20E-016 | 0 | 0.22 | 3.49E-005 | 0.97 | 1.09E-004 | 0.62 | 1.82E-009 | 1.11E-006 | 0 | 1.53E-003 | 8.81E-005 |  |
| significant | ** | no | ** | no | ** | no | ** | no | ** | ** | no | ** | no | ** | no | ** | ** | ** | ** | ** |  |
|  |  |  |  |  |  |  |  |  |  |  |  |  |  |  |  |  |  |  |  |  |  |
| Coevolving | 1029 | 227 | 617 | 752 | 616 | 862 | 343 | 346 | 677 | 852 | 125 | 263 | 454 | 135 | 600 | 327 | 606 | 787 | 97 | 413 | 10128 |
|  | 0.1 | 0.02 | 0.06 | 0.07 | 0.06 | 0.09 | 0.03 | 0.03 | 0.07 | 0.08 | 0.01 | 0.03 | 0.04 | 0.01 | 0.06 | 0.03 | 0.06 | 0.08 | 0.01 | 0.04 |  |
| All-site | 130325 | 24840 | 79354 | 93762 | 62903 | 140860 | 45865 | 65027 | 65038 | 121055 | 28039 | 45652 | 63431 | 32321 | 89684 | 49433 | 88104 | 94874 | 22372 | 54006 | 1396945 |
|  | 0.09 | 0.02 | 0.06 | 0.07 | 0.05 | 0.1 | 0.03 | 0.05 | 0.05 | 0.09 | 0.02 | 0.03 | 0.05 | 0.02 | 0.06 | 0.04 | 0.06 | 0.07 | 0.02 | 0.04 | 1 |
| difference | 0.01 | 0 | 0 | 0.01 | 0.02 | -0.02 | 0 | -0.01 | 0.02 | 0 | -0.01 | -0.01 | 0 | -0.01 | 0 | 0 | 0 | 0.01 | -0.01 | 0 |  |
| difference ratio | 0.09 | 0.26 | 0.07 | 0.11 | 0.35 | -0.16 | 0.03 | -0.27 | 0.44 | -0.03 | -0.39 | -0.21 | -0.01 | -0.42 | -0.08 | -0.09 | -0.05 | 0.14 | -0.4 | 0.5 |  |
| P-value | 0 | 0 | 0.07 | 4.29E-003 | 2.39E-014 | 1.62E-007 | 0.56 | 3.61E-009 | 2.20E-016 | 0.37 | 3.13E-008 | 1.52E-004 | 0.78 | 5.70E-011 | 4.25E-002 | 0.09 | 1.82E-001 | 9.62E-005 | 2.61E-007 | 2.71E-001 |  |
| significant | ** | ** | no | ** | ** | ** | no | ** | ** | no | ** | ** | no | ** | * | no | no | ** | ** | no |  |
| Amino acid | A | C | D | E | F | G | H | I | K | L | M | N | P | Q | R | S | T | V | W | Y |  |
