# Supplementary material for: Coevolution of amino acid residues in the key photosynthetic enzyme Rubisco
Source: BMC Evol Biol. 2011 Sep 23;11:266. doi: 10.1186/1471-2148-11-266 (PMC3190394; doi:10.1186/1471-2148-11-266)
Supplement: Additional file 2 — Positive selection amino acid composition. [file 1471-2148-11-266-S2.DOC]

Appendix 2. Positive selection amino acid composition

| PAML |  |  |  |  |  |  |  |  |  |  |  |  |  |  |  |  |  |  |  |  |  |
| --- | --- | --- | --- | --- | --- | --- | --- | --- | --- | --- | --- | --- | --- | --- | --- | --- | --- | --- | --- | --- | --- |
|  | A | C | D | E | F | G | H | I | K | L | M | N | P | Q | R | S | T | V | W | Y | sum |
| sum | 942 | 137 | 478 | 707 | 364 | 345 | 358 | 612 | 175 | 343 | 198 | 294 | 226 | 217 | 494 | 378 | 412 | 596 | 0 | 253 | 7529 |
| percentage | 0.13 | 0.02 | 0.06 | 0.09 | 0.05 | 0.05 | 0.05 | 0.08 | 0.02 | 0.05 | 0.03 | 0.04 | 0.03 | 0.03 | 0.07 | 0.05 | 0.05 | 0.08 | 0 | 0.03 |  |
| All-site | 130325 | 24840 | 79354 | 93762 | 62903 | 140860 | 45865 | 65027 | 65038 | 121055 | 28039 | 45652 | 63431 | 32321 | 89684 | 49433 | 88104 | 94874 | 22372 | 54006 | 1396945 |
| percentage | 0.09 | 0.02 | 0.06 | 0.07 | 0.05 | 0.1 | 0.03 | 0.05 | 0.05 | 0.09 | 0.02 | 0.03 | 0.05 | 0.02 | 0.06 | 0.04 | 0.06 | 0.07 | 0.02 | 0.04 | 1 |
| difference | 0.03 | 0 | 0.01 | 0.03 | 0 | -0.06 | 0.01 | 0.03 | -0.02 | -0.04 | 0.01 | 0.01 | -0.02 | 0.01 | 0 | 0.01 | -0.01 | 0.01 | -0.02 | -0.01 |  |
| difference ratio | 0.34 | 0.02 | 0.12 | 0.4 | 0.07 | -0.55 | 0.45 | 0.75 | -0.5 | -0.47 | 0.31 | 0.19 | -0.34 | 0.25 | 0.02 | 0.42 | -0.13 | 0.17 | -1 | -0.13 |  |
| P-value | 2.20E-016 | 0.79 | 0.01 | 2.20E-016 | 0.17 | 2.20E-016 | 9.42E-013 | 2.20E-016 | 2.20E-016 | 2.20E-016 | 0 | 0 | 1.53E-010 | 0 | 0.62 | 4.10E-012 | 0 | 0 | 2.20E-016 | 0.02 |  |
| significant | ** | no | * | ** | no | ** | ** | ** | ** | ** | ** | ** | ** | ** | no | ** | ** | ** | ** | * |  |

| Fitmodel |  |  |  |  |  |  |  |  |  |  |  |  |  |  |  |  |  |  |  |  |  |
| --- | --- | --- | --- | --- | --- | --- | --- | --- | --- | --- | --- | --- | --- | --- | --- | --- | --- | --- | --- | --- | --- |
|  | A | C | D | E | F | G | H | I | K | L | M | N | P | Q | R | S | T | V | W | Y |  |
| sum | 855 | 133 | 364 | 485 | 170 | 160 | 204 | 958 | 141 | 849 | 265 | 167 | 234 | 272 | 223 | 952 | 474 | 843 | 39 | 163 | 7951 |
| percentage | 0.11 | 0.02 | 0.05 | 0.06 | 0.02 | 0.02 | 0.03 | 0.12 | 0.02 | 0.11 | 0.03 | 0.02 | 0.03 | 0.03 | 0.03 | 0.12 | 0.06 | 0.11 | 0 | 0.02 |  |
| All-site | 130325 | 24840 | 79354 | 93762 | 62903 | 140860 | 45865 | 65027 | 65038 | 121055 | 28039 | 45652 | 63431 | 32321 | 89684 | 49433 | 88104 | 94874 | 22372 | 54006 | 1396945 |
| percentage | 0.09 | 0.02 | 0.06 | 0.07 | 0.05 | 0.1 | 0.03 | 0.05 | 0.05 | 0.09 | 0.02 | 0.03 | 0.05 | 0.02 | 0.06 | 0.04 | 0.06 | 0.07 | 0.02 | 0.04 | 1 |
| difference | 0.01 | 0 | -0.01 | -0.01 | -0.02 | -0.08 | -0.01 | 0.07 | -0.03 | 0.02 | 0.01 | -0.01 | -0.02 | 0.01 | -0.04 | 0.08 | 0 | 0.04 | -0.01 | -0.02 |  |
| difference ratio | 0.15 | -0.06 | -0.19 | -0.09 | -0.53 | -0.8 | -0.22 | 1.59 | -0.62 | 0.23 | 0.66 | -0.36 | -0.35 | 0.48 | -0.56 | 2.38 | -0.05 | 0.56 | -0.69 | -0.47 |  |
| P-value | 1.35E-005 | 0.48 | 2.26E-005 | 0.03 | 2.20E-016 | 2.20E-016 | 0 | 2.20E-016 | 2.20E-016 | 2.07E-010 | 2.20E-016 | 5.07E-009 | 8.50E-012 | 6.15E-011 | 2.20E-016 | 2.20E-016 | 0.21 | 2.20E-016 | 3.16E-015 | 2.20E-016 |  |
| significant | ** | no | ** | * | ** | ** | ** | ** | ** | ** | ** | ** | ** | ** | ** | ** | no | ** | ** | ** |  |

|  | TOTAL |  |  |  |  |  |  |  |  |  |  |  |  |  |  |  |  |  |  |  |  |
| --- | --- | --- | --- | --- | --- | --- | --- | --- | --- | --- | --- | --- | --- | --- | --- | --- | --- | --- | --- | --- | --- |
|  | A | C | D | E | F | G | H | I | K | L | M | N | P | Q | R | S | T | V | W | Y |  |
| sum | 1629 | 220 | 724 | 1118 | 501 | 479 | 460 | 1398 | 303 | 1060 | 431 | 422 | 432 | 385 | 667 | 1138 | 777 | 1192 | 39 | 382 | 13757 |
| percentage | 0.12 | 0.02 | 0.05 | 0.08 | 0.04 | 0.03 | 0.03 | 0.1 | 0.02 | 0.08 | 0.03 | 0.03 | 0.03 | 0.03 | 0.05 | 0.08 | 0.06 | 0.09 | 0 | 0.03 |  |
| All-site | 130325 | 24840 | 79354 | 93762 | 62903 | 140860 | 45865 | 65027 | 65038 | 121055 | 28039 | 45652 | 63431 | 32321 | 89684 | 49433 | 88104 | 94874 | 22372 | 54006 | 1396945 |
| percentage | 0.09 | 0.02 | 0.06 | 0.07 | 0.05 | 0.1 | 0.03 | 0.05 | 0.05 | 0.09 | 0.02 | 0.03 | 0.05 | 0.02 | 0.06 | 0.04 | 0.06 | 0.07 | 0.02 | 0.04 | 1 |
| difference | 0.03 | 0 | 0 | 0.01 | -0.01 | -0.07 | 0 | 0.06 | -0.02 | -0.01 | 0.01 | 0 | -0.01 | 0 | -0.02 | 0.05 | -0.01 | 0.02 | -0.01 | -0.01 |  |
| difference ratio | 0.27 | -0.1 | -0.07 | 0.21 | -0.19 | -0.65 | 0.02 | 1.18 | -0.53 | -0.11 | 0.56 | -0.06 | -0.31 | 0.21 | -0.24 | 1.34 | -0.1 | 0.28 | -0.82 | -0.28 |  |
| P-value | 2.20E-016 | 0.11 | 0.04 | 4.31E-011 | 1.23E-006 | 2.20E-016 | 0.69 | 2.20E-016 | 2.20E-016 | 6.69E-005 | 2.20E-016 | 0.19 | 3.77E-015 | 0 | 6.79E-014 | 2.20E-016 | 0 | 2.20E-016 | 2.20E-016 | 4.02E-011 |  |
| significant | ** | no | * | ** | ** | ** | no | ** | ** | ** | ** | no | ** | ** | ** | ** | ** | ** | ** | ** |  |
